# Constraint optimization of an integrated production model utilizing history matching and production forecast uncertainty through the ensemble Kalman filter

**DOI:** 10.1038/s41598-024-64213-2

**Published:** 2024-06-12

**Authors:** Mehdi Fadaei, Mohammad Javad Ameri, Yousef Rafiei

**Affiliations:** Department of Petroleum Engineering, Amir Kabir University of Technology, No. 350, Hafez Avenue, Valiasr Square, Tehran, Iran

**Keywords:** History matching, Uncertainty, Constraint optimization, Integrated model, Ensemble Kalman filter, Energy storage, Chemical engineering

## Abstract

The calibration of reservoir models using production data can enhance the reliability of predictions. However, history matching often leads to only a few matched models, and the original geological interpretation is not always preserved. Therefore, there is a need for stochastic methodologies for history matching. The Ensemble Kalman Filter (EnKF) is a well-known Monte Carlo method that updates reservoir models in real time. When new production data becomes available, the ensemble of models is updated accordingly. The initial ensemble is created using the prior model, and the posterior probability function is sampled through a series of updates. In this study, EnKF was employed to evaluate the uncertainty of production forecasts for a specific development plan and to match historical data to a real field reservoir model. This study represents the first attempt to combine EnKF with an integrated model that includes a genuine oil reservoir, actual production wells, a surface choke, a surface pipeline, a separator, and a PID pressure controller. The research optimized a real integrated production system, considering the constraint that there should be no slug flow at the inlet of the separator. The objective function was to maximize the net present value (NPV). Geological data was used to model uncertainty using Sequential Gaussian Simulation. Porosity scenarios were generated, and conditioning the porosity to well data yielded improved results. Ensembles were employed to balance accuracy and efficiency, demonstrating a reduction in porosity uncertainty due to production data. This study revealed that utilizing a PID pressure controller for the production separator can enhance oil production by 59% over 20 years, resulting in the generation of 2.97 million barrels of surplus oil in the field and significant economic gains.

## Introduction

For the effective management of modern oil and gas fields, it is essential to update simulation models by incorporating production data and geological parameterization. It is crucial to update simulation cells in a way that aligns with geological assumptions to maintain a coherent model. The management is increasingly requesting a probabilistic evaluation of different development scenarios. Reservoir models generate distribution functions for key production metrics, such as total oil production, which reflect the uncertainty in reservoir knowledge. It is necessary to regularly and quickly update the models to make well-informed decisions based on the available data.

A separator is an apparatus utilized in oil well operations to separate the oil, water, and gas constituents. The extent of phase separation corresponds to the degree of separation achieved among these constituents. The efficiency of the separator and the magnitude of phase separation directly impact the amount of oil that can be stored in the stock tank.

During oil production, the reservoir’s pressure decreases, resulting in alterations in the hydrocarbon components. To achieve enhanced phase separation, it is crucial to appropriately adjust the dimensions of the separator. However, modifications to the separator’s dimensions are not feasible during production. Therefore, adjusting the separator’s pressure becomes essential in order to optimize phase separation. Additionally, this practice prevents issues such as carry-over, foaming, and emulsion formation, which can negatively impact the production process and pose safety hazards. Consequently, maintaining the separator pressure at the desired value at all times is of utmost importance.

The pipelines are subject to various flow regimes, one of which is slug flow. This flow pattern induces pressure and flow rate fluctuations, which can influence the performance of the separator and its associated equipment^1^. Slug flow is a complex phenomenon that can be further categorized into distinct subtypes based on the characteristics of the gas and liquid phases, including slug length, slug frequency, and slug velocity^1^.

This study uses the EnKF technique to calibrate the actual oil field model and determine the level of uncertainty reduction by assimilating production data. Different ensembles are used to assess the efficiency of EnKF and measure the uncertainty in production predictions with the help of an updated ensemble. This research represents the first application of EnKF to an actual reservoir for history matching and uncertainty analysis of production forecasts. The EnKF methodology is applied in this study to an integrated production model using a real oil reservoir.

**Figure 1:**
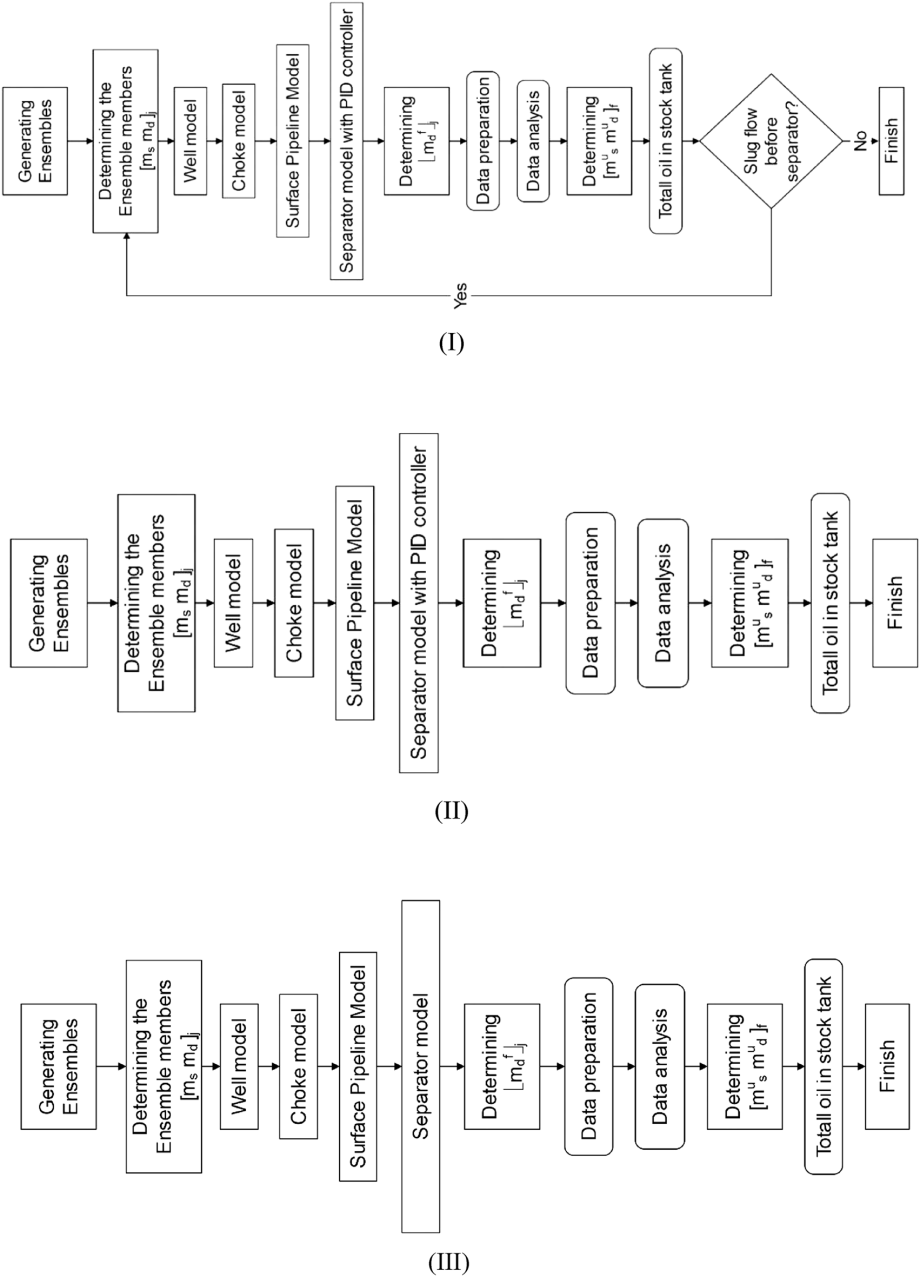
Description of the EnKF workflow with integrating surface facilities. The model state vector, m_d_, controls pressure and saturation, etc., and the model parameter vector, m_s_, controls porosity and permeability.

One of the innovations of this study is the implementation of the Ensemble Kalman Filter (ENKF) method to assess uncertainty in production forecasting. Additionally, this study introduces the integration of the ENKF method with the integrated production model for the first time in the oil industry. In the integrated production model, the ENKF prediction model simulates the behavior of the actual oil reservoir. The models of the well, surface choke, pipeline, and surface separator are interconnected to create a cohesive integrated model. This research proposes three algorithms, as depicted in Fig. 1, to optimize the integrated model. Algorithm I has a single optimization constraint, which is the absence of slug flow before the separator. Algorithm I is employed in the PID controller separator model.

In Algorithm II, there are no additional constraints besides the optimization process. It is also utilized in the PID controller separator model. However, in Algorithm III, there are no constraints in the optimization process, and no PID controller is employed in the separator model. The problem of integrating different sub-models into a unified and coherent integrated model for production optimization is recognized as a promising approach. Furthermore, the three algorithms presented in Fig. 1 are compared in terms of functionality and efficiency to enhance oil production in the storage tank and eliminate the slug flow regime. Identifying the most effective model that can increase oil storage and simultaneously eliminate the slug flow regime before the separator represents a novel approach. Moreover, this research introduces the constraint optimization of the integrated model and the simultaneous adjustment of the ENKF method with the separator PID controller, which is a unique contribution in its field.

This study is divided into three sections. The first section focuses on history matching, the second section on assessing uncertainty in production forecasts, and the third section on production estimation over 20 years using three algorithms. These algorithms are integrated models that include the reservoir, wells, chokes, surface pipelines, and separator with a PID pressure controller. In this section, we will compare our research to similar studies and highlight the benefits of our study.

The Ensemble Kalman Filter (EnKF) is a Monte Carlo-based technique that is commonly employed for calibrating oceanographic models. It is widely utilized for historical matching and uncertainty estimation in reservoir simulation due to its essential features. EnKF is especially appropriate for real-time simulations as it combines generated data with available equivalent models^2^.

EnKF uses a collection of Gaussian models that are updated linearly to store the most recent production data. EnKF can be utilized in conjunction with any reservoir simulator that can restart, and it does not necessitate gradients for history-matching or sensitivity coefficients^3^.

EnKF utilizes a set of Gaussian models that are linearly updated to store the latest production data. It can be used alongside any reservoir simulator that can restart, and it does not require gradients for history-matching or sensitivity coefficients^3^.

Nasima et al.^4^ conducted a study in which they utilized the ENKE method to update both static parameters, such as porosity and permeability, and dynamic variables to align with real-time production data. The Norne Oil Field was divided into three ensembles, specifically referred to as Case A, Case B, and Case C. Following the simulation using EnKF, all three cases yielded precise porosity ranges.

In this study, we utilized a real reservoir model and combined the reservoir, well, chokes, pipeline, and separator sub-models into a cohesive production system. Additionally, we employed three algorithms, considering certain constraints, to optimize production to maximize the NPV and eliminate slug flow before the separator.

Byeongcheol et al.^5^ conducted a study that demonstrated the utility of ensemble-based analyses in comparing equiprobable scenarios of reservoir models. The researchers proposed a specific preprocessing method for selecting good initial models, which effectively reduced the ensemble size. Subsequently, they employed EnKF to stochastically predict production performances. The study specifically focused on two 3D models and found that EnKF yielded reliable assimilation results while significantly reducing computation time. To create a production system, a realistic reservoir model was combined with models of the well, chokes, pipeline, and separator. In addition, three algorithms with specific constraints were utilized to optimize production, maximize net present value (NPV), and eliminate slug flow before the separator.

Rajabi-Kochi et al.^6^ proposed an integrated model for the PUNQ-S3 reservoir located in the North Sea. The authors conducted a sensitivity analysis to determine the key parameters affecting the target functions. By employing a two-level Plackett–Burman design, they were able to identify 16 parameters with the highest impact. Subsequently, they narrowed this selection down to seven variables that exhibited the greatest influence on the target functions, namely net present value, cumulative oil production, and cumulative water production. To establish the proxy model, a three-level Box-Behnken experimental design was employed for each target function. This design effectively took into account the interactions between the variables. The suitability and reliability of the proxy model for each target function were validated based on the decision variables. Lastly, a multi objective optimization was carried out with the objective of maximizing net present value and cumulative oil production, while minimizing cumulative water production. This optimization process utilized a parameter known as composite desirability.

AlRassas et al.^7^ propose a new hybrid intelligence time series model for forecasting oil production in two distinct oil fields in China and Yemen. This model, named AO-ANFIS, is a modified version of the Adaptive Neuro-Fuzzy Inference System (ANFIS) that incorporates a novel optimization algorithm called the Aquila Optimizer (AO). The AO algorithm draws inspiration from the behavior of Aquila in nature. The performance of the AO-ANFIS model was evaluated using real-world datasets obtained from local partnerships. Additionally, a comprehensive competitor analysis was conducted to gain insights into the market landscape. A comparative analysis was performed between the AO-ANFIS model, the traditional ANFIS model, and several modified ANFIS models that utilize different optimization algorithms. The numerical results and statistics provide evidence of the superiority of the AO-ANFIS model over both the traditional ANFIS model and the modified models. Furthermore, the results demonstrate that the AO algorithm significantly enhances the prediction accuracy of the ANFIS model. Consequently, the AO-ANFIS model can be regarded as an efficient tool for analyzing time series data.

Akter et al.^8^ discussed the challenges of using the EnKF algorithm for joint state-parameter estimation. They made two modifications to the algorithm and created a benchmark problem, known as the ‘tank series model’, to test its effectiveness. The researchers then applied a similar approach to a nonlinear two-dimensional reservoir that was undergoing water flooding operation to assess its performance in history matching. Additionally, they conducted a sensitivity analysis to further evaluate the algorithm’s effectiveness. In our study, we used a realistic reservoir model and integrated it with models of the well, chokes, pipeline, and separator to construct a unified production system. Furthermore, we utilized three algorithms with specific constraints to optimize production, maximize the net present value (NPV), and eliminate slug flow before the separator.

Xuechen et al.^9^ introduced a novel framework that employs a Bidirectional Gated Recurrent Unit (Bi-GRU) and Sparrow Search Algorithm (SSA) to improve the precision of oil rate prediction. The Bi-GRU effectively integrates historical and future information within production sequences and associated characteristics. The SSA is employed to optimize the hyperparameters of the Bi-GRU model. In order to evaluate the feasibility, reliability, and efficiency of the proposed approach, three scenarios were conducted, consisting of an ideal single well from the simulation model, an actual single well under varying production constraints, and multiple actual wells. The performance of the model was compared to that of traditional decline curve analysis, conventional time series methods, and one-way recurrent neural networks. The findings indicate that the proposed approach surpasses the others in terms of accuracy and reliability.

In their study, Wang et al.^10^ conducted research on the development of deep belief network (DBN) models to accurately and effectively predict the production performance of unconventional wells. To ensure the construction of a comprehensive training database, the researchers ran 815 numerical simulation cases and employed the Bayesian optimization algorithm to optimize the hyperparameters of the network model. The results revealed that the DBN models surpassed traditional machine-learning techniques, such as back-propagation (BP) neural networks and support vector regression (SVR), in terms of prediction accuracy and generalization ability. Moreover, Wang et al.^10^ utilized the trained DBN model to optimize fracturing design, yielding impressive outcomes. The model demonstrated instantaneous and accurate predictions of the production performance of unconventional wells, and its reusability establishes it as a valuable tool for optimizing fracturing designs. This research lays a solid foundation for anticipating the production performance of unconventional reservoirs and provides valuable insights into the development of data-driven models for energy conversion and utilization.

Liu et al.^11^ developed a model that efficiently predicts oil production by employing an ensemble empirical mode decomposition (EEMD)-based Long Short-Term Memory (LSTM) learning paradigm. In their study, the researchers initially divided the original oil production series into a training set and a test set. The test set data was progressively integrated into the training set and decomposed using EEMD to obtain multiple intrinsic mode functions (IMFs). The stability of these IMFs was assessed based on their means and curve similarity, determined through Dynamic Time Warping (DTW). The most stable IMFs were subsequently chosen as predictor variables for machine learning. By considering the trend and contextual information of the production series, LSTM was employed to establish a predictive model for production forecasting. The optimal hyperparameters for LSTM were determined utilizing the Genetic Algorithm (GA). To validate and evaluate the proposed model, data from two real oilfields in China were utilized. The empirical results demonstrated that the proposed approach yielded extremely accurate production forecasts.

Chen et al.^12^ proposed an efficient workflow for evaluating the uncertainty of optimal well rates in waterflood problems. Their research is highly commendable and presents several innovative contributions. Specifically, they developed a flow feature clustering method using streamline and unsupervised machine learning techniques to reduce the number of geologic realizations required for representing geologic uncertainty. This approach significantly enhances the workflow's efficiency.

In their study, Chen et al.^12^ employed a set of historical production and injection data. Initially, they generated an ensemble of history-matched geologic realizations using the ensemble smoother with multiple data assimilation (ESMDA) technique. Subsequently, they utilized streamline time of flight (TOF) and principal component analysis (PCA) to extract the flow features from all realizations. Based on these features, they employed the k-means clustering algorithm to derive a subset of realizations that represent the entire ensemble. Given the exceptional nature and comprehensiveness of Chen et al.’s^12^ work, this article aims to follow their methodology. Moreover, this research endeavors to incorporate the separator controller in the constraint optimization process to prevent the occurrence of slug flow regimes before the separator.

Ren et al.^13^ conducted a study on a waterflood field consisting of more than 1000 wells. They found that using modern field management techniques with full-fidelity 3D geo-cellular reservoir models posed computational challenges. To address this issue, Ren et al.^13^ developed a new flow-network data-driven model called GPSNet, which allowed for rapid history matching and optimization. The researchers utilized Ensemble Smoother with Multiple Data Assimilation (ESMDA) to minimize errors during the history matching process. Subsequently, a best-matched candidate was selected for numerical optimization to maximize oil production rates, while also considering field conditions. The implementation of GPSNet in the waterflood field resulted in excellent history-matching outcomes at the field level, as well as satisfactory matches for key producers. Thus, the successful application of GPSNet demonstrates its potential as a quick and reliable decision-making tool for reservoir management.

Ren et al.^13^ introduced an effective workflow for reservoir management in waterflood scenarios. Their research is highly commendable and offers several innovative contributions. Additionally, this study strives to integrate the separator controller into the constraint optimization process, aiming to prevent slug flow regimes prior to the separator and maximize total oil production in the stock tank.

The novelty of this study lies in its utilization of three algorithms simultaneously, each serving a specific purpose: production optimization, slug flow removal, and production forecast uncertainty with ENKF. Algorithm I employs a separator equipped with a PID pressure controller and two constraints. These constraints aim to maximize the NPV (Net Present Value) and eliminate the slug flow regime before the separator. In contrast, Algorithm II focuses solely on maximizing the NPV, utilizing a separator with only one constraint. Lastly, Algorithm III does not feature a PID pressure controller nor any constraints for the separator. These algorithms encompass integrated models that encompass the actual oil reservoir, wells, chokes, surface pipelines, and a real oil field separator.

## Modelling

This section presents a comprehensive explanation of integrated system modeling.

### The ensemble Kalman filter (EnKF)

The EnKF is a statistical method used to solve inverse problems involving sequential time data. It achieves this by employing multiple-state vector realizations to quantify model uncertainty. Additionally, it uses a state vector to represent both observations and parameters during the modeling process.

In reservoir simulation, the EnKF updates a group of reservoir models sequentially as production data is assimilated. The reservoir's state vector consists of three parameters: static factors, dynamic factors, and production data. Static factors, such as porosity and permeability, remain constant throughout the simulations and are typically utilized in conventional history-matching processes. On the other hand, dynamic factors, including cell pressure, gas, and water saturation, and GOR, are used in flow simulations. Additionally, production rates, bottom-hole pressure, water cut, and gas–oil ratio observations are considered for these variables. Multiple realizations are used to model the state variables^8^.

1 $$y_{k, j} - \left[ {\begin{array}{*{20}c} {m_{s} } \\ {m_{d} } \\ d \\ \end{array} } \right] = 0$$

At time $t_{k}$, the *j*th ensemble member of the state vector is represented by $y_{k, j}$. $m_{s}$ and $m_{d}$ denote the static and dynamic variables respectively, while d is the production data vector. EnKF updates a group of reservoir models with current production data, resulting in an updated ensemble with explicit model uncertainty statistics. The filter has two processes: prediction and analysis. The prediction procedure involves the simulation of each model until the following observation date, whereas data assimilation and state variable updating are included in the analysis process. The state variables are progressed over time in the following way:

2 $$y_{k, j}^{f} - F\left( {y_{k - 1, j}^{u} } \right) = 0, \quad j = 1, \;2, \ldots ,\; N_{e}$$

F is the forward model, $y_{k, j}^{f}$ is the *j*th state vector after the kth forecast, and $N_{e}$ is the number of ensemble members. The “f” superscript represents the output of the simulator before the updating of the Kalman Filter. The matrix H_k_, is the Jacobian matrix where H and K represent, respectively, the observation operator and the gain matrix and it does not need to be derived explicitly from the non-linear equations, so it is in the form^14^:

3 $$H_{k} - \left[ {{\mathbf{0}}|I} \right] = {\mathbf{0}}$$

**0** is a $N_{d,k} \times (N_{y,k} - N_{d,k} )$ matrix with all 0’s as entries; I is a $N_{d,k} \times N_{d,k}$ identity matrix. The covariance matrix $C_{d,k}$ is of dimension $N_{d,k} \times N_{d,k}$ and is diagonal if the production data errors are independent. The ensemble of forecasted results $(y_{k, j}^{f} )$ can be used with the statistical method to estimate the covariance for the state variables at the time $t_{k}$, which is defined by the matrix $C_{y,k}^{f}$^15^.

4 $$C_{y,k}^{f} - \frac{1}{{N_{e} - 1}}\mathop \sum \limits_{j = 1}^{{N_{e} }} (y_{k, j}^{f} - \overline{y}_{k}^{f} )\;(y_{k, j}^{f} - \overline{y}_{k}^{f} )^{T} = 0$$

The Kalman gain is a parameter that determines the significance of the measurements and current-state estimate in the filtering process. It can be adjusted to optimize the filter's performance for specific requirements. Increasing the gain leads to a greater emphasis on the most recent measurements, resulting in a more responsive adaptation to them^16^. The Kalman gain $K_{k}$ can be computed using the following equation, where $y_{k, j}^{f}$ is the *j*th ensemble member of the forecasted state variable, which is a vector of dimension $N_{y,k}$.

5 $$K_{k} - C_{y,k}^{f} H_{k}^{T} (H_{k} C_{y,k}^{f} H_{k}^{T} + C_{d,k} )^{ - 1} = 0$$

Assuming the Gaussian distribution of $y_{k,j}$, update state vector with variance minimizing scheme using Kalman gain as a weighting matrix with observed production data, $d_{k, j}$. In particular, the state variables are transformed by weighting them with the Kalman gain matrix, $K_{k}$.

6 $$y_{k, j}^{u} - y_{k, j}^{f} - K_{k} \left( {d_{k,j} - H_{k} y_{k,j}^{f} } \right) = 0$$

The update to the forecast vector (u) is determined by the discrepancy between the simulated and observed production data. Larger discrepancies lead to greater adjustments to the original state vector. Afterward, the covariance matrix can be calculated.

7 $$C_{y,k}^{u} = \left( {I - K_{k} H_{k} } \right)C_{y,k}^{f}$$

The reservoir model was calibrated using the Kalman filter, incorporating one year of production data (the time resolution of the data is daily). The calibrated model was then integrated with the well, choke, pipeline, and separator model. The EnKF workflow was described, and sub-models for the reservoir, well, choke, surface pipeline, and separator were solved simultaneously, while also considering the constraint of no slug flow before the separator, as depicted in Fig. 1.

Update the covariance matrix for the current observations until new ones become available. Equations (5) and (6) apply to a Gaussian distribution when the errors in the model and observations are uncorrelated. The Ensemble Kalman Filter (EnKF) maintains spatial correlations by using initial variograms for nonlinear models. The original geological interpretation remains unchanged. Figure 1 shows three flowcharts that predict field production over 20 years.

### The real oil reservoir

The paper discusses an onshore oil field in Iran that includes a 45-foot sand body and a shale layer cap as its actual oil reservoir. Initially, the reservoir was saturated with gas and had a thin layer of oil. A geophysical analysis was conducted, which identified the gas-oil contact in the field. This analysis involved three appraisal wells and two production wells. Initially, the gas-oil contact was found at a total depth of 8344.3 feet TVD, while the water–oil contact was identified at 9213 feet. The field also contained seven layers of sand with porosity log values ranging from 9 to 18%, and no vertical flow barriers were observed. A black-oil model was used to simulate the field.

**Figure 2:**
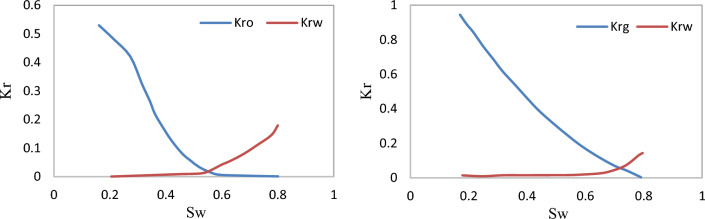
The relative permeability curves for oil–water (left), and gas–oil (right).

The oil gravity, oil formation volume factor, oil viscosity, and undersaturated compressibility was 35° API, 1.4 RB/STB, 0.29 cp and s 1.34 × 10^−5^ *psia*^−1^, respectively. Also, initial bubble point: 3885 psi, GOR: 1 Mscf/STB in oil rim. The simulation grid was 160 × 80 × 25 cubic cells, with a dimension of 390 ft × 390 ft × 390 ft. 304,000 cells were active. The graph in Fig. 2 shows the relative permeability for gas–water and oil–water systems.

#### Baseline geological model

**Figure 3:**
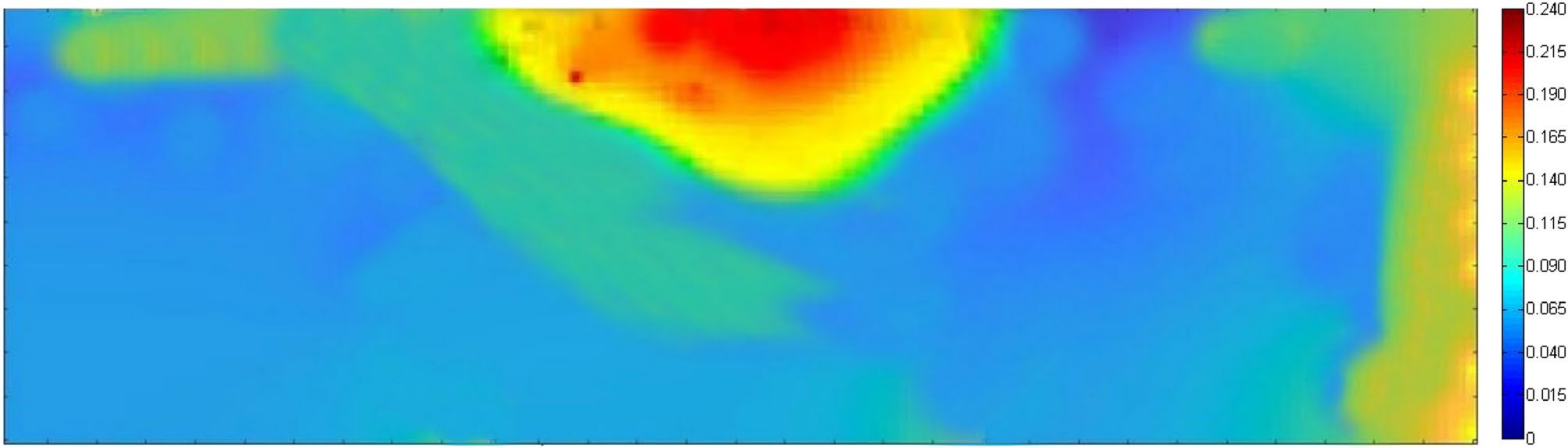
Porosity map of oil field layer. Red indicates high porosity (up to 24%), and violet indicates very low (down to 2%).

To accurately simulate the reservoir, it was necessary to determine the porosity values for the simulation grid. Three wells were used to create a baseline model with log porosity values. Kriging was then employed to expand these values throughout the reservoir. Figure 3 displays a representative layer's Kriged porosity map.

The use of the same spacing for both geological and simulation grids facilitated the easy transfer of porosity values. Upon analyzing the data, we were able to establish a deterministic relationship between permeability and porosity. A permeability ratio of 1 was used to define a static parameter. Saturation levels are initiated at the endpoints of relative permeability.

### Gaussian ensembles generation

The EnKF updates Gaussian models in an ensemble sequentially. SGSim generates simulation models in groups to produce correlated porosity fields using isotropic bi-dimensional variograms with a range of 4000 m. Unlike Yusuf et al.^17^, the porosity distribution in this study was restricted to observed porosities in appraisal wells.

A total of 135 porosity fields were created using stochastic modeling for the EnKF. These fields were divided into three ensembles: A, B, and C, with 40, 100, and 130 members, respectively. The impact of different ensemble sizes was assessed, with 100 members being the commonly used size based on experience in atmospheric sciences^18^.

A large ensemble is necessary to accurately estimate field uncertainty, while a smaller ensemble is sufficient for matching production data. For a reasonable history match, a small reservoir model found solutions with 40 ensemble members^8^. On the other hand, a 100-member ensemble is enough for a reasonable history match in the PUNQ-S3 model. A small group is necessary for a balanced solution, while larger groups are needed to measure uncertainty in estimated static fields^19,20^.

### Sub-models correlations

The Duns and Ross correlation, along with its flowchart^21^, was utilized as a well sub-model. Its output is then directed to the choke sub-model, which determines critical and sub-critical flows. The output of the choke sub-model is subsequently employed as input for the pipeline sub-model^22^. The pipeline sub-model utilized the Beggs–Brill correlation^23^. The surface pipelines had a total length of 1335 m and a diameter of 4 inches.

To optimize the performance of the gas–liquid separator, a pressure controller is employed. This controller ensures that the separator operates under optimal conditions by estimating, adjusting, and controlling its performance. This section presents the equations utilized by the separator pressure controller. Achieving optimal operational conditions for the separator is vital to maximizing the amount of oil in the stock tank. Key parameters include the oil formation volume factor, gas-oil ratio (GOR), and the weight of oil in the stock tank.

#### Equations of separator pressure PID controller

The PID controller equations for separator pressure control in this section were derived. Equation (8) relates gas pressure, inlet and outlet gas, and liquid flow rates^24^.

8 $$\frac{dP}{{{\text{dt}}}}{\text{V}}_{{\text{G}}} - {\text{RT}}\frac{{{\uprho }_{{\text{G}}} }}{{{\text{M}}_{{\text{G}}} }}\left( {{\text{q}}_{{{\text{G}},{\text{ in}}}} - K_{v} \alpha \frac{P}{SG}} \right) - {\text{P}}\left( {{\text{q}}_{{{\text{L}},{\text{ in}}}} - {\text{q}}_{{{\text{L}},{\text{ out}}}} } \right) = 0$$

where ${\text{q}}_{{{\text{G}},{\text{ in}}}}$ is inlet gas volume flow rate $\left[ {\frac{BBL}{{Day}}} \right]$, R is 8.314 $\frac{J}{mol.K}$, P is separator pressure [psi], $V_{G}$ real gas velocity $\left[ \frac{ft}{S} \right]$, $M_{G}$ is the molar weight of the gas $\left[ \frac{g}{mol} \right]$, α is the Percentage of the valve opening, and T is the Gas temperature [F]. Equation (8) is a control equation and is simplified to $\dot{X} - a.X - b.U$ = 0 The coefficients a, and b are $\frac{{q_{L, in} - q_{L,out} }}{{V_{G} }} - \frac{{K_{v} aRT\rho_{G} }}{{V_{G} M_{G} SG}}$ and $\frac{{RT\rho_{G} }}{{V_{G} M_{G} }}$ respectively. A first-order filter was added to the PID controller for noise reduction. The optimal value of the filter’s time constant was determined through trial and error, as shown in Eq. (9).

9 $$K_{P} - K(s) + \frac{{K_{I} }}{s} + \frac{{K_{d} s}}{{T_{f} s + 1}} = 0$$

The Ziegler–Nicholas optimization method improved the performance of the PID controller. Table 1 shows the control parameter values after using the tuning method.

**Table 1:** Optimal values of the PID controller parameters.

State variable	*t*_*c*_ (s)	*K*_*P*_	*K*_*d*_	*K*_*I*_	*T*_*f*_
Pressure	5	0.075	− 0.2	4	2

The control variables of the PID controller were the gas flow rate. The upper and lower limits of control signals were equal to 1 and 0 m^3^ s^−1^, respectively. The rate of change according to the activation dynamics was equal to 0.05 m^3^ s^−2^.

## History matching the oil field by using ENKF

The coded EnKF was utilized to match the history of the oil reservoir model by employing state vectors that encompassed both static and dynamic variables. Data including bottom hole pressures, well production rates, WCT, and GOR were employed to estimate production. Ensembles A, B, and C were analyzed to assess the impact of changes in ensemble size. Moving forward, we will examine production data and discuss misfit errors.

### Production data for EnKF based history matching

**Figure 4:**
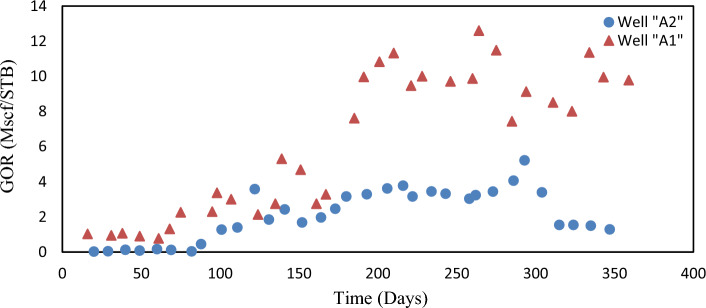
- The measured GOR data for wells A1 (represented by triangles) and A2 (represented by circles).

**Figure 5:**
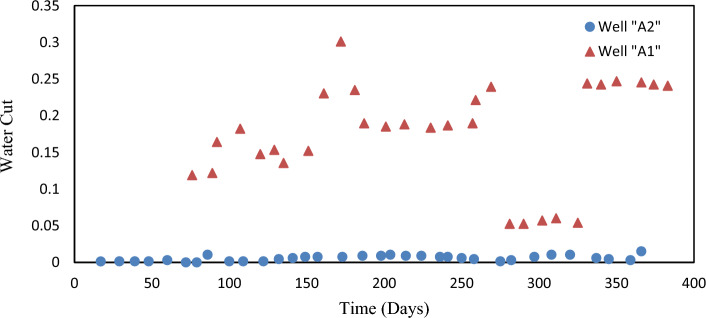
- The measured WCT data for wells A1 (represented by triangles) and A2 (represented by circles).

There were two vertical oil wells named A1 and A2 in the oil field. During the history-matching period, GOR and WCT targets were simulated for a year, and actual data was evaluated. Figures 4 and 5 display the history of both wells. Well A2 was mainly operated under dry conditions.

As mentioned previously, Fig. 5 displays the measured water contact (WCT) data for wells A1 and A2. High GOR values are generally considered to be more reliable than low GOR values. Similarly, trends that exhibit a consistent increase are typically more precise than those that oscillate. In the specific case of wells A1 and A2, it can be observed that the GOR of well A1 is more reliable compared to that of well A2. However, it is important to note that the decline in the GOR of well A2, from 5 to 2.5 MScf/STB, and the extremely low WCT values (< 0.05) observed for well A1 between day 275 and day 340, were deemed to be unreliable. Table 2 presents the errors utilized for the weighting of observations in the oil field. For a more comprehensive understanding, please refer to Table 3, which displays the errors or standard deviations utilizing production data.

**Table 2:** The used errors for observations weighting of the oil field.

Measuring parameter	Well name	
	A1	A2
Gas–oil-ratio^1^	0.35 MScf/STB for GOR values ≥ 5.5 MScf/STB, 0.25 MScf/STB for lower values	0.55 MScf/STB for GOR values gathered till day 290, 590% for later time values
Water cut^2^	0.45 for values outside the low-reliability time window, and 590% inside	0.0024 all the data

^1^GOR.

^2^WCT.

**Table 3:** The average values of the objective functions for the three ensembles before and after EnKF integration.

Ensemble	Average objective function	
	Before EnKF	After EnKF
A	568.4	153.9
B	596.5	135.4
C	598.4	136.6

### Analysis of the results of EnKF history matching

**Figure 6:**
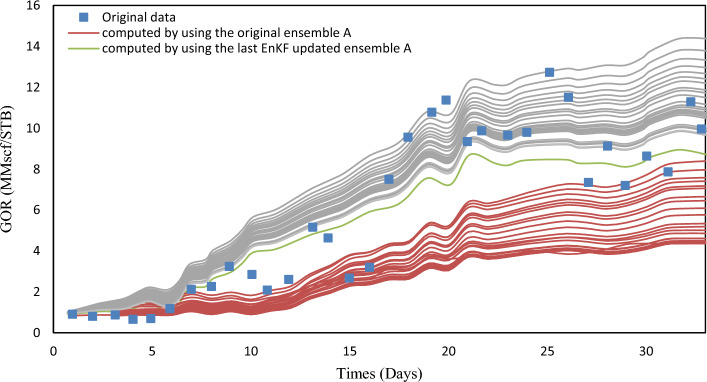
Observed values for A1 GOR.

**Figure 7:**
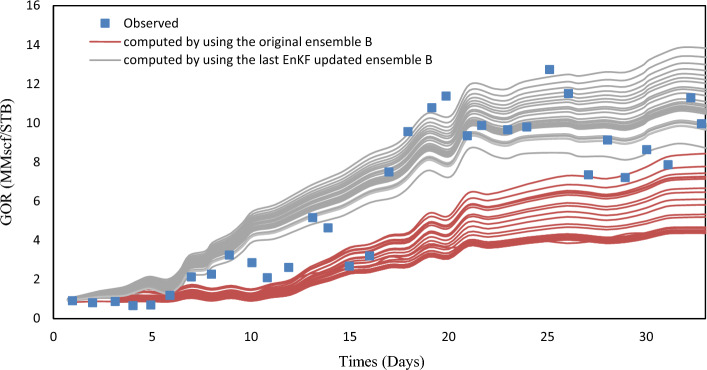
Observed values for A1 GOR.

**Figure 8:**
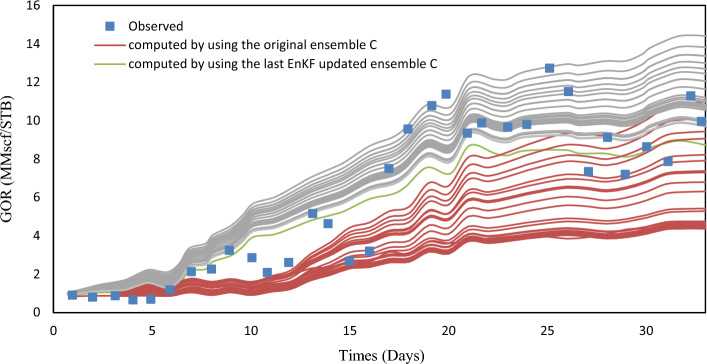
Observed values for A1 GOR.

EnKF was used for history matching on A, B, and C ensembles with 33 assimilation periods. EnKF effectiveness can be assessed by observing the evolution of forecasted quantities like $y_{k, j}^{f}$ in real-time scenarios. EnKF calibration improves short-term predictive value in real-time applications and ocean modeling. To evaluate the effectiveness, porosity fields from the previous assimilation step were used to simulate the history period for one year. Figures 6, 7, and 8 show the comparison of EnKF porosity in blue and original porosity in red for ensembles A-C, with observed data for Well A1 GOR.

The Ensemble Kalman Filter (EnKF) algorithm has shown promising results as a tool for history-matching. The assimilation process effectively adjusts the simulated data envelope to closely match the observed trend, while also reducing the variability of the simulated values. Similar patterns were noticed in the well A2 GOR and the WCT, which resemble the well A1 WCT. To better assess the performance of history matching, one can calculate the ensemble-averaged objective function J.

10 $$J_{A} - \frac{1}{{2N_{e} }}\mathop \sum \limits_{j = 1}^{{N_{e} }} \mathop \sum \limits_{i = 1}^{{N_{obs} }} \left( {\frac{{o_{i} - c_{ij} }}{{\sigma_{i} }}} \right)^{2} = 0$$

**Figure 9:**
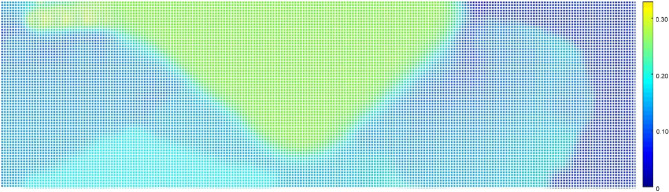
Average original porosity for ensemble A in a representative layer.

The equation involves the observed values of gas-oil ratio (GOR) and water cut (WCT), represented by $o_{i}$ and $o_{j}$, respectively, for two wells. The value $c_{ij}$ corresponds to the observed value $o_{i}$ and is obtained through the simulation of the Jth ensemble member. Table 3 defines standard deviations. Before and after EnKF integration, Figs. 7, 8, 9 show the average objective function values of three ensembles. EnKF systematically decreases objective function values, with significant improvement for larger ensembles.

### Statistical analysis of integrated ensembles

The EnKF updates the covariance and mean of the ensemble by using Monte Carlo sampling of the posterior probability. This method analyzes the effects on ensemble statistics by using the mean and standard deviation as estimators.

### Ensemble mean updates

To define the ensemble mean, an average porosity field $\langle {m_{s} } \rangle_{a}^{o/u}$ was computed by taking the mean value of the porosity field across all members of the ensemble, where

11 $$\langle {m_{s} } \rangle_{a}^{o/u} = \frac{1}{{N_{e} }}\mathop \sum \limits_{j = 1}^{{N_{e} }} m_{s, j}^{o/u}$$

Equation (11) offers two choices for the mean-field calculation. The first one is based on the original ensemble (denoted by superscript ‘o’), while the second one is based on the ensemble after the EnKF updates (denoted by superscript ‘u’). The approximations of the Kriged baseline porosity field are represented by the average porosity fields $\langle {m_{s} } \rangle^{o}$, which can be observed in both Figs. 9 and 3.

**Figure 10:**
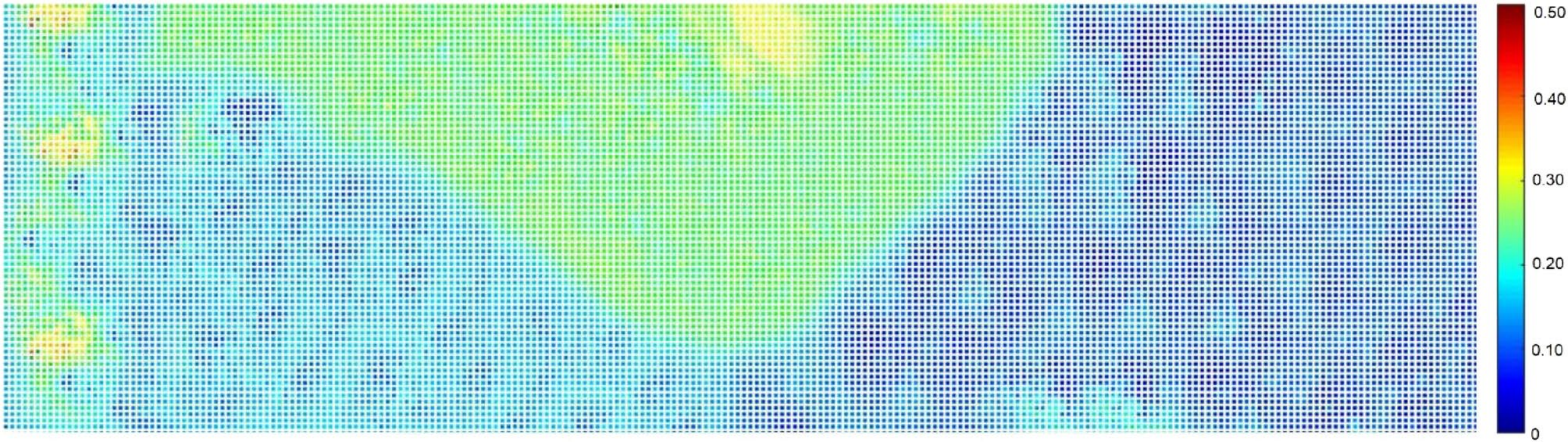
Average porosity for ensemble A after EnKF integration in a representative layer.

Equation (11) computes the mean field using original or updated ensembles (labeled A, B, or C). The average porosity fields $\langle {m_{s} } \rangle^{o}$ approximate the Kriged baseline porosity field, as seen by comparing the original ensemble A’s average porosity field (Fig. 10) with the Kriged porosity map (Fig. 9).

Porosity fields, denoted by $\langle {m_{s} } \rangle_{a}^{u}$, provide unbiased estimates of the posterior mean. Figure 1 displays porosity maps for ensemble A. The EnKF integration led to an increase in the average porosity in the West and South-East flanks. Ensemble C had similar porosity values to the original mean. The lack of porosity over-shooting may indicate more consistency with larger ensembles. Over-shooting did occur in the far West, but it did not affect oil-in-place computations; it only provided pressure support.

The integration of EnKF increased the average porosity in the West and South-East flanks. Ensembles A and B showed high porosity values of up to 42%, while ensemble C’s porosity values were similar to the original mean field. Overshooting mainly occurred in the far West, away from the oil rim, but did not negatively affect oil-in-place computations. In this framework, the best models to represent the integrated ensembles are those defined by the ensemble means, namely $\langle {m_{s} } \rangle_{A}^{u}$, $\langle {m_{s} } \rangle_{B}^{u}$, and $\langle {m_{s} } \rangle_{C}^{u}$.

**Figure 11:**
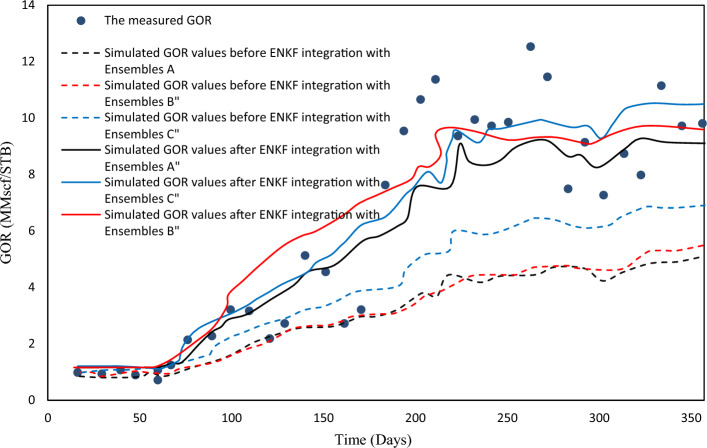
The measured GOR values for Well A1.

In Fig. 11, black, red, and blue lines represent ensembles A, B, and C, respectively. Figure 11 shows GOR values computed based on integrated porosity mean values $\langle {m_{s} } \rangle_{A}^{u}$, $\langle {m_{s} } \rangle_{B}^{u}$, and $\langle {m_{s} } \rangle_{C}^{u}$, together with original porosity mean values $\langle {m_{s} } \rangle_{A}^{o}$, $\langle {m_{s} } \rangle_{B}^{o}$, and $\langle {m_{s} } \rangle_{C}^{o}$. This image shows that the mean models can also be considered as individually history-matched models, which are appropriate for deterministic purposes.

As anticipated, in a scenario where the computing system is held equal, an increase in the number of ensemble members results in a corresponding increase in computing time. Figure 11 displays the computing time for Ensembles A, B, and C prior to the integration of ENKF, over a forecast period of approximately one year, which was measured at 320, 465, and 504 min, respectively. Conversely, after the integration of ENKF, the computing time for Ensembles A, B, and C over the same forecast period increased to 768, 1048, and 1123 min, respectively.

### Analysis of uncertainty for production forecasting

According to Table 3, it is evident that the optimal number of ensembles is 100 (Ensemble B). This is due to the fact that the average value of the cost function after integration is lower for Ensemble B compared to Ensembles A and C, at the minimum possible state. Therefore, Ensemble B was exclusively utilized to forecast production for a period of 20 years, as it demonstrated superior performance when compared to Ensembles A and C.

**Figure 12:**
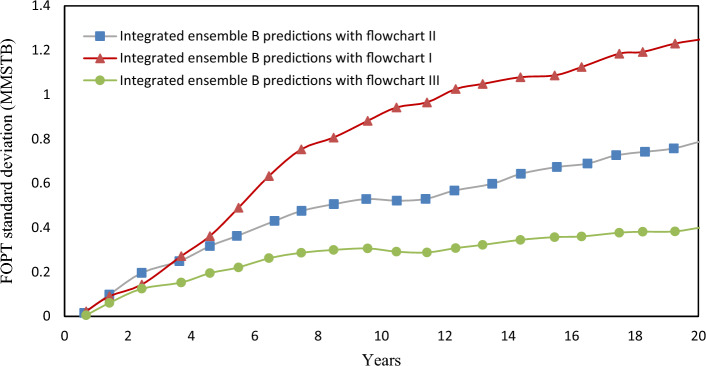
The standard deviation of cumulative oil production vs. time for 20 years forecast, the original ensemble B predictions, and integrated ensemble B predictions.

To assess the uncertainty of production forecasts, Ensemble B can generate predictions for 20 years. Both wells have a minimum tubing head target of 400 psi, and if the water cut (WCT) exceeds 50%, abandonment constraints come into play. Flowcharts I and II serve to restrict slug flow before reaching the separator. The implementation of the Ensemble Kalman Filter (EnKF) resulted in a 39% reduction in the standard deviations of predicted cumulative oil production by the end of the 20th year, as depicted in Fig. 12.

**Figure 13:**
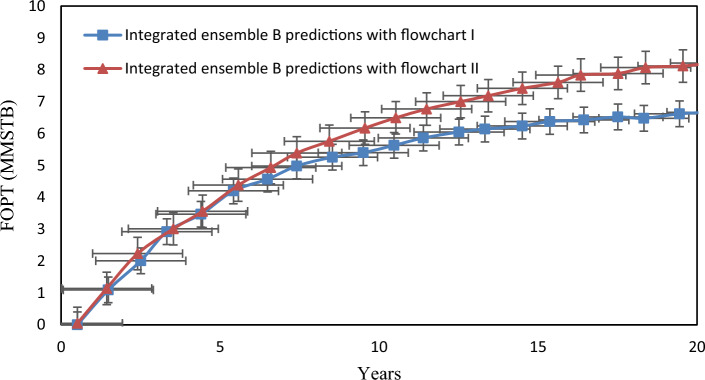
The average value of FOPT during the 20-year forecast for ensemble B.

In Fig. 13, the integrated ensemble predictions exhibit statistically significant differences from the original predictions. This is evidenced by the combined average incremental cumulative oil production and standard deviations.

In the figure above, the error bars were defined using the standard deviations. The Table 4 displays the estimated quantity of oil production predicted using flowcharts. According to Table 3, Ensemble B with 100 members performed the best and was selected.

**Table 4:** The estimated oil production over 20 years for three flowcharts using ensemble B after EnKF integration.

Ensemble	Flowchart	Estimated oil production over 20 years of production (MMSTB)
B	I	6.85
	II	7.98
	III	5.01

According to the results presented in Table 4, Flowchart II, which only uses a pressure controller, produces more oil after 20 years than Flowchart I. However, Flowchart I, which utilizes both a separator pressure controller and slug flow control, can prevent slug flow despite producing less oil. To enhance oil production, an integrated production model can be employed, incorporating the use of a Kalman filter, a separator's pressure controller, and sludge control. This approach has the potential to increase oil production by at least 59%. By implementing this method, approximately 2.97 million barrels of surplus oil can be produced in 20 years for the actual oil field, resulting in improved production control and a significant boost in economic income.

## Comparison of the functionality between the provided code and commercial integrated modeling software

In this section, we will compare the capabilities of the code presented in this research with existing commercial software for integrated production modeling. For instance, when discussing the capabilities of the provided code in comparison with the software provided in The IPM suite, we can highlight that the code provided in this research, unlike The IPM suite, is capable of measuring the level and pressure of the separator at any given time. It can also measure and control production using appropriate control models based on the optimization process. Furthermore, this code can optimize the entire integrated production system in real time, alleviate the slug flow regime prior to the separator, and simultaneously adapt itself with predictive Kalman filter models, which surpasses the capabilities of commercial software for integrated production system modeling such as The IPM suite, Petroleum Experts (PetEx) suite, and Olga.

## Conclusions

The EnKF method was used to adjust porosity fields to simulate the production of an oil reservoir for nearly a year. The results confirmed that EnKF can be used for history matching in real reservoir models. Additionally, an analysis was conducted to determine how the effectiveness of EnKF is affected by the number of fields included in the statistical ensembles. For this purpose, three ensembles consisting of 40, 100, and 130 members were used.

This study attempted to explore the relationship between the effectiveness of EnKF and the size of the ensemble for a real problem. However, the limited number of ensembles analyzed prevented us from reaching any definitive conclusions on key topics, such as the number of fields required from a practical standpoint. Nevertheless, some interim conclusions can be drawn.

According to this analysis, using ensembles consisting of 100 or 130 members would improve the calibration quality. This was confirmed through the objective function values reported in Table 3, although the differences were not significant from a quantitative perspective.

This study is the first attempt to couple EnKF with an integrated model consisting of a real oil reservoir, real production wells, a surface choke, a surface pipeline, a separator, and a pressure controller.

In this research, a real integrated production system has been optimized for the first time. The optimization was done by considering the constraint that there is no slug flow at the inlet of the separator, and the objective function was to maximize the net present value (NPV) in the storage tank.

After EnKF integration, mean porosity values showed some local overshooting in the case of ensembles A and B, but not in ensemble C. However, integrated porosity ensembles proved to be useful in predicting production with less uncertainty in ensemble B.

Flowchart II produces more oil in 20 years than Flowchart I. An integrated production model with a Kalman filter, separator pressure controller, and slug control can improve oil production by at least 59%. This approach can generate about 2.97 million barrels of surplus oil in the oil field, resulting in significant economic gains.

## Data Availability

Yes, I have research data to declare. All data will be provided upon request through email (ameri@aut.ac.ir).
